# Conductive PPy@cellulosic Paper Hybrid Electrodes with a Redox Active Dopant for High Capacitance and Cycling Stability

**DOI:** 10.3390/polym14132634

**Published:** 2022-06-28

**Authors:** Shuaishuai Yang, Xueren Qian

**Affiliations:** 1 School of Chemistry and Chemical Engineering, Anshun University, Anshun 561000, China; yangshuaishuai10@163.com; 2 Key Laboratory of Bio-Based Material Science & Technology, Ministry of Education, Northeast Forestry University, Harbin 150040, China

**Keywords:** CFs, PPy, AQS, doping effect, electrochemical performance

## Abstract

Polypyrrole@cellulose fibers (PPy@CFs) electrode materials are promising candidates in the energy storage. Various strategies have been pursued to improve their electrochemical performance. However, the poor conductivity, specific capacitance, and cyclic stability still hindered its application. Compared with the previous studies, we selected AQS with electrochemical activity as a dopant to improve these defects. It exhibits a high capacitance of 829.8 F g^−1^ at a current density of 0.2 A g^−1^, which is much higher than that of PPy@CFs electrode material (261.9 F g^−1^). Moreover, the capacitance retention of PPy:AQS/p-PTSA@CFs reaches up to 96.01% after 1000 cycles, indicating superior cyclic stability. Therefore, this work provides an efficient strategy for constructing high-performance electrode materials for energy storage.

## 1. Introduction

As the global economy experiences rapid growth, humans are facing a shortage of fossil fuels and increasingly hazardous environmental pollution [1,2,3,4]. It is important and urgent, therefore, to develop highly efficient, clean, and renewable energy storage devices. Among them, supercapacitors (SCs) have some remarkable qualities, including fast charging/discharging and long cycle-life [5,6,7]. Unfortunately, their low energy density (20 Wh·Kg^−1^), in comparison to batteries (75 Wh·Kg^−1^), hinders their wide application [8,9].

Conductive polymers have been studied, owing to their distinctive properties, since they were discovered in 1977 [10]. Polypyrrole (PPy) has gained much attention as one of the most common conductive polymers due to its conductivity, pseudo-capacitive performance, and ease of synthesis for application in sensors, catalysis, energy storage and conversion, corrosion protection, and electromagnetic interference shielding [11,12,13,14,15,16,17,18,19]. Furthermore, based on its superior pseudo-capacitance, researchers have made many attempts to incorporate it in the energy storage field. However, its conductivity and machinability remain challenges. To overcome the limitation of machinability, recent efforts have focused on the flexible substrates, such as cellulose fibers (CFs), textile, polymer, etc. [20,21,22]. Among these substrates, CFs has the characteristics of rich sourcing, environmental friendliness, sustainability, light weight, low price, recyclability, and good flexibility [23,24]. The characteristics of CFs are expected to be applied to flexible energy storage devices. On the other hand, the previous studies of PPy mainly focused on the effects of different types of dopants on the electrochemical behaviors, such as the inorganic anions with small volume of Cl^−^, SO_4_^2−^, NO^3−^, ClO_4_^−^, and PO_4_^3−^ [25], which are easy to be undoped, and results in the destruction of the PPy structure. The other kinds of dopants mainly included aromatic p-methylbenzene sulfonic acid, sulfosalicylic acid, sodium lignosulfonate, sodium dodecyl sulfonate, and other organic macromolecular anions [26]. Nevertheless, one of the defects of dopants, as described above, is that they do not have electrochemical activity, which virtually increases the quality of the electrode, commonly known as “dead mass,” which is not conducive to the electrode materials exerting their electrochemical properties in the charging/discharging process. In addition, there is also a class of sulfonate macromolecular dopants of anthraquinone, which contains oxidation/reduction functional groups with electrochemical activity in their molecular structure, showing potential value in the field of electrochemistry. However, its poor conductivity has impeded its widespread application in energy storage.

Therefore, in this study, we designed cellulose-based electrode material with optimized PPy via the in situ method. Anthraquinone sulfonate (AQS) dye molecule is selected as the dopant, and the sulfonic group (–SO_3_^−^) functional group in its molecule is tested to see whether it can be used to optimize the electrochemical performance of PPy. This doping strategy will introduce dye molecules with oxidation/reduction functional groups into the PPy molecular chain. On the one hand, it will improve the conductivity of dye molecules. On the other hand, dye molecules will also have a positive impact on the conductivity and specific capacitance of PPy. This “synergy” between the two will help them give full play to their electrochemical characteristics and promote their application in the field of electrochemistry. 

## 2. Materials and Methods

### 2.1. Materials and Reagents

Canada market bleached softwood kraft pulp was provided by Mudanjiang Hengfeng Paper Co., Ltd. (Heilongjiang, China) and was beaten to 37 ^o^SR before use. The pyrrole monomer was purchased from Tianjin Zhiyuan Chemical Reagent Co., Ltd. (Tianjin, China). The AQS was purchased from Shanghai Aladdin Biochemical Technology Co. Ltd. (Shanghai, China). The ferric chloride (FeCl_3_·6H_2_O) was purchased from Shanghai Macklin Biochemical Co., Ltd. (Shanghai, China). The sulfuric acid (H_2_SO_4_) was purchased from Xilong Chemical Co., Ltd. (Yulin, China).

### 2.2. Preparation of PPy:AQS/p-TSA@CFs Composites

The PPy:AQS@CFs composite was prepared via in situ oxidative polymerization under ice-bath. Typically, the CFs of 0.5 g (dry weight) were dispersed in distilled water under vigorous stirring for 30 min to obtain the CFs suspension, and a certain amount of P-TSA (0, 1, 2, 3 and 4 mmol), AQS, and py monomer were added until dissolved. Afterwards, the APS was added, drop by drop, as an oxidant to initiate the polymerization reaction for 6 h under ice-bath. Subsequently, the mixture was washed with distilled water several times to remove oligomers and other impurities. Finally, the composites was obtained and named PPy:AQS@CFs, PPy:AQS/p-TSA-1@CFs, PPy:AQS/p-TSA-2@CFs, PPy:AQS/p-TSA-3@CFs, and PPy:AQS/p-TSA-4@CFs. For comparison, the PPy@CFs composites also were prepared by the same method.

### 2.3. Characterizations

The micromorphology and elemental mapping were investigated via SEU 8010 scanning electron microscope (SEM). The resistivity of the sample was measured by RTS-8 four-probe tester. The test process used the thin disc test method. The setting current is calculated by measuring the thickness, diameter, and probe spacing, and the resistivity of the sample is finally obtained. The X-ray diffraction (XRD) patterns were performed using the X’ Pert3 Powder XRD system with a scan rate of 2° min^−1^ at a range of 5° to 40°. Fourier transform infrared spectroscopy (FT-IR) was recorded on a Bruker Vertex 80 V Infrared Emission spectrometer with a scanning range of 4000–400 cm^−1^ at a resolution of 4 cm^−1^. The X-ray photoelectron spectroscopy (XPS) was obtained by Thermo ESCALAB 250XI. The RTS-8 four-probe tester was employed to measure conductivity. 

### 2.4. Electrochemical Measurements

The electrochemical measurements (CV, GCD, and EIS) of all electrode materials were performed in 0.6 M H_2_SO_4_ on the CHI-660E workstation. The Ag/AgCl in a 1 M KCl, Pt plate and CFs-based electrodes (1 × 1.5 cm^−2^) were the reference electrode, counter electrode, and working electrode, respectively. The test of cyclic stability was conducted accord to the methods described in the literature [27]. The specific capacitance of electrode materials was calculated under the three-electrode system via the equation:

$$Cs=\frac{I\Delta t}{m\Delta V}$$

where *C*s are specific capacitance (F g^−1^) for GCD profiles, and m, *I*, Δ*V* and Δ*t* represent active mass (g), current density (A g^−1^), potential window, and discharge time (s), respectively.

## 3. Results and Discussion

### 3.1. Fabrication Process

**Figure 1:**
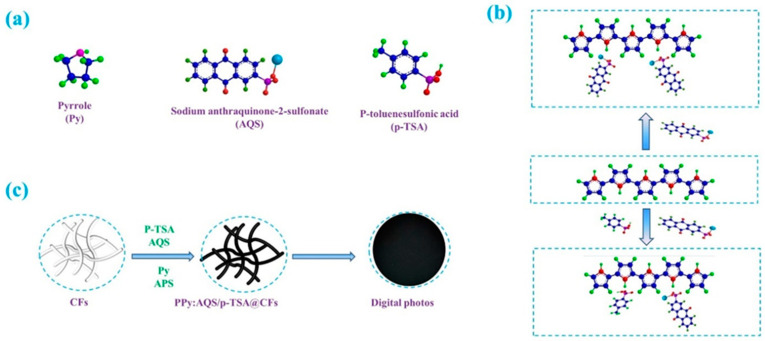
Ball-and-stick models of monomer Py, dopants AQS, and p-TSA, (**a**); doping mechanism diagram of PPy:AQS/p-TSA@CFs electrode materials, (**b**); and schematic diagram of the preparation process of PPy:AQS/p-TSA@CFs paper electrode materials, (**c**).

Figure 1a shows the bat model of pyrrole, AQS, and p-TSA. Based on the doping mechanism of PPy, the p-TSA with acidity and AQS with electrochemical activity were selected as dopants to improve the conductivity and capacitive performance of PPy@CFs electrode materials. The doping mechanism and preparation process paper electrode materials are shown in Figure 1b,c. Among these, the sulfonic acid group in the AQS molecular structure plays a doping role in PPy, while the benzoquinone structure as an active component enhances the capacitance of the PPy:AQS/p-TSA@CFs electrode materials. The addition of p-TSA plays the role not only of dopant, but also as surfactant. The preparation conditions and the conductivity of the PPy:AQS/p-TSA@CFs paper electrode materials are shown in Table 1. The introduction of p-TSA has an positive impact on the conductivity of the PPy:AQS@CFs paper electrode materials. When its dosage is 2 mmol, the paper electrode material exhibits the best conductivity of 34.06 S m^−1^.

### 3.2. Characterization Analysis

**Figure 2:**
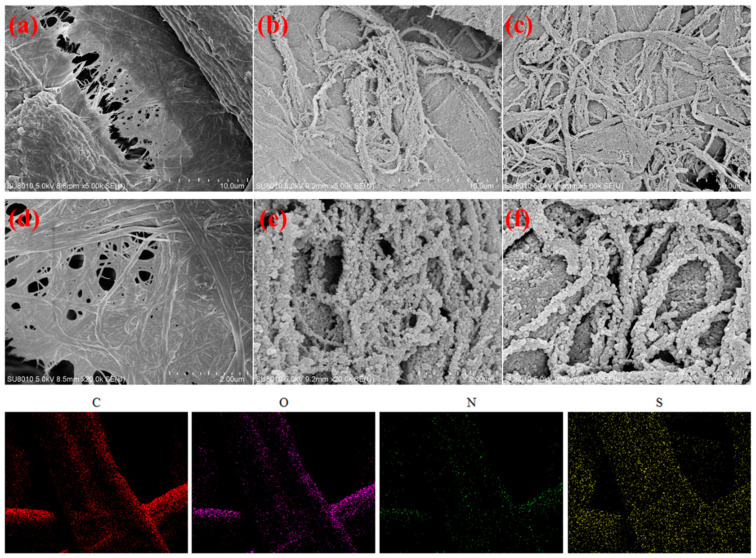
SEM images of CFs (**a**,**d**), PPy@CFs (**b**,**c**), and PPy:AQS/p-TSA@CFs (**e**,**f**) electrode materials and EDS element analysis (**C**, **O**, **N**, and **S**) images of PPy:AQS/p-TSA@CFs electrode materials.

The surface morphology of the CFs, PPy@CFs, and PPy:AQS/p-TSA@CFs electrode materials was performed via SEM. It can be observed that the surface of the CFs (Figure 2a,d) was clean and smooth, whereas the morphology of the PPy@CFs and PPy:AQS/p-TSA@CFs electrode materials showed a rough interface, which proved the deposition of PPy on the surface of the CFs. However, the morphology of the PPy@CFs and PPy:AQS/p-TSA@CFs electrode materials also exhibited differences. When there is no AQS and p-TSA, the PPy with spherical particles dispersed on CFs, and their distribution is relatively loose. Interestingly, using the AQS and p-TSA as dopants and surfactants increased the dispersion of pyrrole in the system, which is conducive to the uniform loading of polypyrrole on the surface of CFs. In parallel, based on the doping mechanism of PPy, the PPy:AQS/p-TSA@CFs electrodes also possessed superior conductivity. From Figure 2, the distribution of C, O, N, and S can be observed for the PPy:AQS/p-TSA@CFs electrode materials, which also proved the uniform dispersion of doped PPy on CFs.

**Figure 3:**
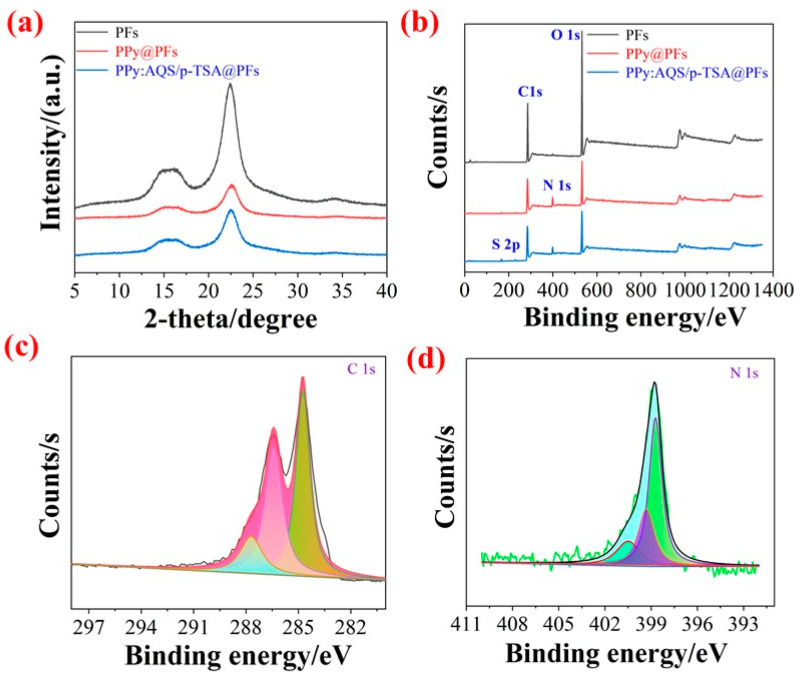
XRD (**a**) and XPS (**b**) images of CFs, PPy@CFs, and PPy:AQS/p-TSA@CFs; XPS C 1s (**c**) and N 1s (**d**) high-resolution spectra of PPy:AQS/p-TSA@CFs paper electrode materials.

Figure 3a illustrated the XRD patterns of CFs, PPy@CFs, and PPy:AQS/p-TSA@CFs composites. From the position of the peaks, there is no significant difference between the three. The peaks at 15.6° and 22.5° in the XRD pattern correspond to the (110) and (200) crystal planes of CFs [28,29]. By comparison, there are no additional diffraction peaks for the PPy@CFs, and PPy:AQS/p-TSA@CFs electrode materials, but the strength of the crystal plane (200) decreases, which may be due to the combination of PPy and CFs [30]. The loading of PPy on the surface of CFs does not change the position of the diffraction peaks, indicating that the introduction of PPy does not affect the crystal structure of the matrix material, which is due to the amorphous characteristics of PPy, and its diffraction peak is about between 20° and 30° [31], which also overlaps with the (200) crystal plane of the CFs [32,33,34].

XPS is one of the effective methods used to analyze the phase composition and valence states of elements. As shown in the Figure 3b, the XPS spectra of the CFs only show the C and O elements, while the XPS spectra of the PPy@CFs and PPy:AQS/p-TSA@CFs composites reveal the extra N and S peaks, along with the extra, respectively. The presence of the N and S peaks suggests the deposition of doping PPy with AQS and p-TSA. In order to further analyze the composition of the electrode materials, the XPS C 1s and N 1s high-resolution spectrum of the PPy:AQS/p-TSA@CFs composites is shown in Figure 3c,d. The C 1s can be fitted into three peaks at 284.7, 286.4, and 288.2 eV. Among these, the peak at 284.7 eV is ascribed to C–C, C–H, and C=C, the peak at 284.7 eV belongs to C–OH, C–N, and =C–N^+^ [35], and the peak at 288.2 eV is attributed to C–C, C–H, and C=O. From the N 1s high-resolution spectrum, the peaks can be fitted to =N– (398.5 eV), –NH– (399.3 eV), and –NH^+^ (400.6 eV), respectively. All the results prove the loading of PPy, and the existence of NH^+^ suggests oxidative PPy in the composites, which is very important to improve the conductivity of electrode materials, and also promote the formation of more significant capacitive properties [36].

**Figure 4:**
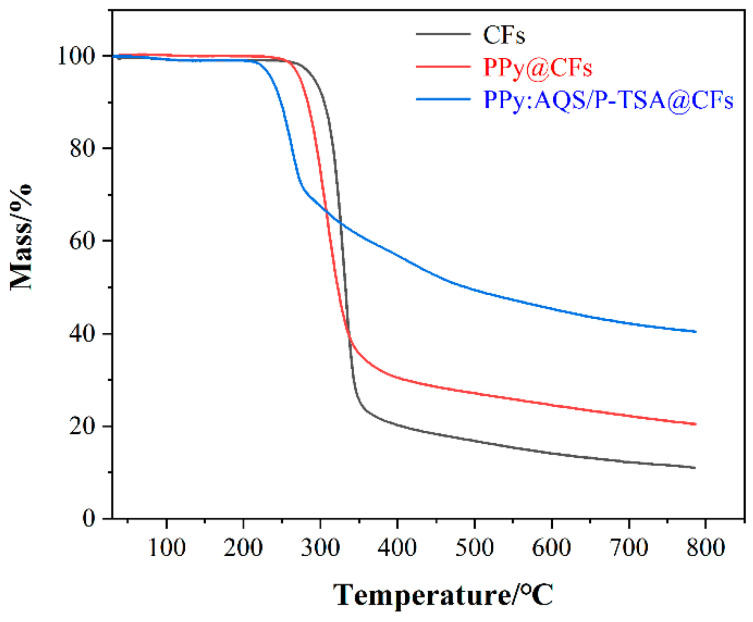
Thermogravimetric curves of CFs, PPy@CFs, and PPy@AQS/p-TSA@CFs.

The thermal stability of the material is also extremely important for the composite. In this paper, the thermal stability of the material is characterized using a TGA test at 30~800 °C, and the results are shown in Figure 4. It can be seen from the figure that the thermal weight loss rate of the three at 800 °C is CFs > from large to small PPy@CFs > PPy:AQS/p-TSA@CFs; the thermal stability is the opposite, i.e., PPy:AQS/P-TSA@CFs. The composite has the best thermal stability. At the same time, it also can be proved that the PPy are loaded successfully onto the CFs.

### 3.3. Electrochemical Performance

**Figure 5:**
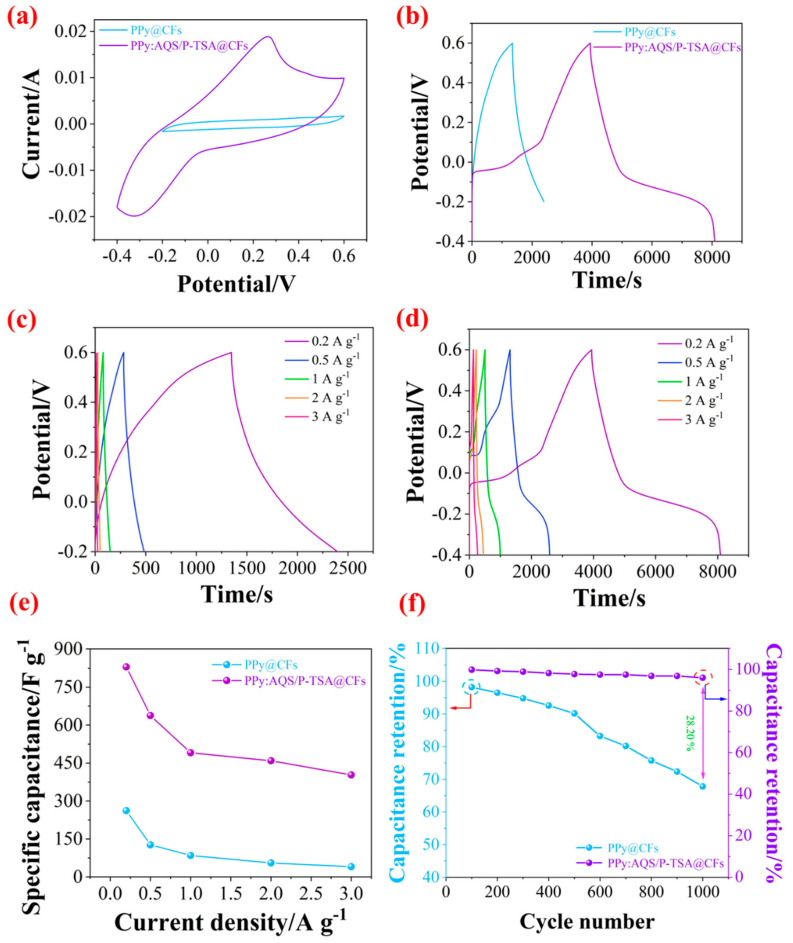
(**a**) CV curves of PPy@CFs and PPy:AQS/p-TSA@CFs electrode materials at a scan rate of 5 mV s^−1^; (**b**) GCD curves of PPy@CFs and PPy:AQS/p-TSA@CFs electrode materials at a current density of 0.2 A g^−1^; (**c**,**d**) GCD curves of PPy@CFs and PPy:AQS/p-TSA@CFs electrode materials at different current densities (0.2, 0.5, 1, 2, and 3 A g^−1^); (**e**) specific capacitance of PPy@CFs and PPy:AQS/p-TSA@CFs electrode materials at different current densities (0.2, 0.5, 1, 2, and 3 A g^−1^); and (**f**) capacitance retention of PPy@CFs and PPy:AQS/p-TSA@CFs electrode materials in the 1000 cyclic charging/discharging process.

The PPy has attracted extensive attention because of its non-toxicity, high conductivity, and reversible oxidation/reduction pseudocapacitance. Based on the doped conductive mechanism of PPy, the research on dopants plays an extremely important role. Hence, AQS with oxidation/reduction activity was selected as the dopant in the paper. Figure 5a,b exhibits CV and GCD curves of PPy@CFs and PPy:AQS/p-TSA@CFs electrode materials at a scan rate of 5 mV s^−1^ and current density of 0.2 A g^−1^. It can be observed that the obvious oxidation/reduction peaks in the CV curve of the PPy:AQS/p-TSA@CFs electrode material, and compared with the CV curve of the PPy@CFs electrode material, which has a larger closed area, suggesting better capacitive performance. Besides, the GCD curves of both confirm the opinion that the PPy:AQS/p-TSA@CFs electrode material shows a longer discharge time, indicating that it has a higher specific capacitance. The GCD curves of the PPy@CFs and PPy:AQS/p-TSA@CFs electrode materials at various densities of 0.2, 0.5, 1, 2, and 3 A g^−1^ are presented in Figure 5c,d. According to the formula of C_s_, the specific capacitance of the PPy@CFs and PPy:AQS/p-TSA@CFs electrode materials were obtained, as shown in Figure 5e. The specific capacitance of PPy@CFs electrode material are 261.9, 126.6, 84.8, 55.3, and 40.5 F g^−1^, and the specific capacitances of the PPy:AQS/p-TSA@CFs electrode material are 829.8, 637.9, 490.7, 459.2, and 403.2 F g^−1^, respectively. It can be seen that the doped paper electrode material shows more significant capacitive performance, which is due to the doping effect of the AQS dopant and its electrochemical activity.

Cyclic stability is one of the important indexes to evaluate the advantages and disadvantages of electrode materials; the capacitance retention of the PPy@CFs and PPy:AQS/p-TSA@CFs electrode materials under 1000 charge/discharge is shown in Figure 5f. With the continuous cyclic charge/discharge of electrode materials, its specific capacitance continues to decline. This phenomenon can be attributed to the continuous expansion/collapse of the PPy molecular chain during the cycle. However, the capacitance retention of PPy:AQS/p-TSA@CFs with AQS as a dopant is 96.01% after 1000 cycles, which is much higher than that of the PPy@CFs electrode material of 67.81%. The superiority is due to the immobilization of AQS in the PPy chain because of a larger molecular structure, which is more conducive to the structural stability of the electrode material in the electrochemical process. Therefore, the PPy:AQS/p-TSA@CFs electrode material shows better cycle stability.

**Figure 6:**
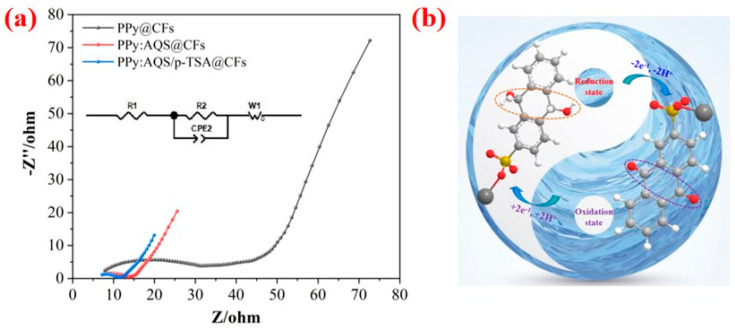
(**a**) Nyquist plots of PPy@CFs, PPy:AQS@CFs, and PPy:AQS/p-TSA@CFs, and (**b**) the mechanism of AQS in the electrochemical process for PPy:AQS/p-TSA@CFs electrode materials.

To investigate the charge transfer and ion diffusion performance of CFs-based electrode materials, we carry out the electrochemical impedance spectroscopy (EIS), and simulate the equivalent circuit according to electrochemical impedance spectroscopy (EIS), which mainly includes three parts, i.e., equivalent series resistance (ESR), charge transfer resistance (Rct), and Warburg diffusion (W). From Figure 6a, we see that the resistance value of PPy@CFs (~8.0 Ω) is higher than that of PPy:AQS@CFs (~7.7 Ω) and PPy:AQS/p-TSA@CFs (~7.2 Ω). Compared with the PPy@CFs and PPy:AQS@CFs, the PPy:AQS/p-TSA@CFs exhibits more significant ion diffusion, which suggests that AQS and p-TSA used as dopants can improve the electrochemical performance. However, as is commonly known, traditional dopants, such as Cl^−^ and SO_4_^2−^, have certain defects. First, they are doped into the molecular chain of a conductive polymer during polymerization, and will be de-doped from the polymer during electrochemical reduction. The further charging and discharging process of the polymer involves the migration of ions in and out of the electrolyte. Second, they have no electrochemical activity, and they will increase the quality of the electrode and reduce the efficiency of the electrode material. The advantage of the AQS dopant has been confirmed for the conductivity, specific capacitance, and cyclic stability of the PPy:AQS/p-TSA@CFs electrode material. Among these, the improvement of conductivity is due to the higher doping level of PPy under the action of AQS. The increase in specific capacitance is due to the presence of the functional groups with electrochemical activity in the molecular structure of AQS. The improvement of cyclic stability can be attributed to the relatively larger molecular structure of AQS, which is fixed in the internal structure of AQS after doping to inhibit the molecular expansion/collapse of PPy caused by the insertion/removal of dopants in the electrochemical process. Therefore, the PPy:AQS/p-TSA@CFs electrode material has higher cycle stability. Figure 6b exhibits the mechanism of AQS in the electrochemical process for the PPy:AQS/p-TSA@CFs electrode materials. The AQS, with active functional groups, can undergo reversible conversion of the oxidized and reduced state, which can provide additional specific capacitance for the electrode material to obtain higher specific capacitance.

## 4. Conclusions

The traditional dopant does not have electrochemical activity, which will virtually increase the invalid mass of electrode materials and is not conducive to its capacitive performance. Therefore, this paper investigated the doping effect of AQS with electrochemical activity on PPy. Firstly, it can improve the doping level of PPy to obtain higher conductivity. Secondly, the introduction of AQS can effectively improve specific capacitance and cyclic stability. The specific capacitance of the PPy:AQS/p-TSA@CFs electrode material is 829.8 F g^−1^ at a current density of 0.2 A g^−1^, which is much higher than that of the PPy@CFs electrode material (261.9 F g^−1^). Meanwhile, the capacitance retention of 96.01% after 1000 cycles is also much higher than that of the PPy@CFs electrode material. The strategy of using AQS as a dopant is simple, convenient, and efficient to obtain more significant electrode material, which may have a potential application in the energy storage field.

## Figures and Tables

**Table 1:** Preparation conditions and conductivity of the PPy:AQS/p-TSA@CFs electrode materials.

Samples	AQS mmol	p-TSA mmol	Py mL	n(APS)/n(Py)	Conductivity S m^−1^
PPy:AQS@CFs	2	0	0.25	1	20.12
PPy:AQS/p-TSA-1@CFs	2	1	0.25	1	25.13
PPy:AQS/p-TSA-2@CFs	2	2	0.25	1	34.06
PPy:AQS/p-TSA-3@CFs	2	3	0.25	1	31.01
PPy:AQS/p-TSA-4@CFs	2	4	0.25	1	23.07

## Data Availability

The authors confirm that the data supporting the findings of this study are available within the article.
